# “We All Have Strengths”: A Retrospective Qualitative Evaluation of a Resilience Training for Latino Immigrants in Philadelphia, PA

**DOI:** 10.1089/heq.2019.0070

**Published:** 2019-10-30

**Authors:** Jamile Tellez Lieberman, Krystal Lobban, Zujeil Flores, Kristin Giordano, Emily Nolasco-Barrientos, Yoshiaki Yamasaki, Ana P. Martinez-Donate

**Affiliations:** ^1^ Department of Community Health and Prevention, Dornsife School of Public Health, Drexel University, Philadelphia, Pennsylvania.; ^2^ The Philadelphia AIDS Consortium (TPAC), Philadelphia, Pennsylvania.

**Keywords:** Latino immigrants, resilience, evaluation, adversity, community resilience

## Abstract

**Background:** Limited research has explored sources of resilience for Latino immigrants or the potential of resilience-based interventions to promote Latino immigrant health and well-being.

**Purpose:** To evaluate Latino immigrants' experiences with a resilience training and application of the training to participants' personal lives and their communities among Latino immigrants.

**Methods:** We conducted a retrospective, qualitative study in Philadelphia, PA from 2017 to 2018. We completed semi-structured, key informant interviews with nine participants who had taken the resilience training, and one facilitator (*N*=10). Transcripts were analyzed via interpretive content analysis.

**Results:** The training resonated deeply with participants because of their personal traumas and immigration-related adversity. Participants were primed by past experiences of violence, as well as by daily struggles they encounter as Latino immigrants in the United States amid worsening anti-immigrant rhetoric and policy. The training was found to be transformative by allowing participants to discover and tap into their own inherent resilience. Participants utilized the knowledge and skills acquired from the training to better manage daily situations, as well as worked to strengthen others within their networks.

**Conclusions:** Resilience-based interventions can help to strengthen communities against adversity. Cultivating resilience in Latino immigrants can have positive effects on psychosocial health. Resilience-building approaches could be implemented as stand-alone or enhancing components of more complex health promotion interventions. More research is needed on resilience, as well as its utility in community-based interventions to promote the health and well-being of Latino immigrants.

## Introduction

Resilience is the ability to withstand adversity by activating coping mechanisms and leveraging assets and resources.^1,2^ It has been described as a trait or outcome but the emerging scholarly consensus is that resilience is a process, dependent on circumstance and context.^3^ Resilience theory offers a conceptual framework with which to characterize the processes that promote the adaptability of individuals and their communities in the face of social disadvantage. The concepts of resilience and community resilience have their public health origins in disaster preparedness, where community resilience is the community's ability to withstand and recover from a disaster, such as pandemics, extreme weather, or terrorism.^4^ Resilience at the community level concerns not just the population itself but also the environment or place in which their resilience is tested.^5,6^ The field of public health is charged with the dismantling of social systems that create health inequalities and disparities. It is impossible to address health inequity without talking about social change and, to some extent, resilience, since resiliency speaks to the vulnerability and survival of communities faced with adversity.^7^

In the United States, some minority groups are more vulnerable to adversity. Latinos in the United States are among the fastest growing ethnic minority groups. The Centers for Disease Control and Prevention estimates that there are 54 million Latinos residing in the United States.^8^ This group is expected to increase to comprise about 25% of the nation's population by 2050.^9^ Latinos in the United States experience serious disparities in health. In particular, migration is a socio-structural factor linked to inequitable health outcomes for Latino immigrants. Immigration exposes these communities to health risks, such as poverty, low education, dangerous working and living conditions, acculturative stress, discrimination, and restricted access to health care.^10^ For example, about 9 million Latinos in the United States are undocumented.^11^ Lack of documentation is a unique barrier to health services and opportunities that positively affect health since access to insurance and health services offered by the government and other organizations is often restricted.^12^ Further, the Trump administration's crack down on Latino immigrant families at the border and nationwide has created an environment of fear and hopelessness.^10,13^ These hostile circumstances constitute an emergency for the Latino immigrant community and heighten the need for preparedness and fortification against adversity. Resilience-based interventions may represent a novel approach to address social determinants of health and mitigate health disparities and inequity for Latino immigrants in the United States during this crisis.

Although the number of studies on resilience is increasing, a few studies examine this concept in Latino immigrants.^14,15^ There is also limited research on sources of resilience for Latinos and on resilience-based interventions for this group.^16,17^ To our knowledge, the majority of resilience trainings in the literature are geared toward special sub-populations of providers who experience trauma, such as police officers, soldiers, and doctors; existing resilience interventions for community members focus primarily on youth and adolescents in a clinical setting.^15–17^ This formative, community-academic study sought to explore the acceptability and potential of resilience-based interventions for this population by qualitatively evaluating a small-scale community resilience program developed by and for Latino immigrants. We were interested in understanding participants' motivations for participating in the program and their experiences of becoming “trained” in resilience. This study also examined the various impacts of the program on the participants themselves, as well as their communities, as recounted by the participants. Finally, the study sought to gather and share implications for the development of community-based interventions that seek to promote the resilience in Latino immigrants.

## Methods

### Study design and background

This retrospective, qualitative study was conducted in Philadelphia, PA from 2017 to 2018 and represented a collaboration between a local academic institution and a local non-profit organization. About 14% of Philadelphia residents are Latino, and one out of every five is foreign-born.^18^ Latinos and Latino immigrants in the city experience the highest rates of poverty compared with other demographic groups, high unemployment and uninsurance rates,^19^ and they are disproportionately burdened by binge drinking, opioid-related deaths, homicidal deaths, mental health illness diagnosis, and new HIV diagnosis.^20^ In response to these health challenges, a training was developed to cultivate resilience among Latino immigrants.

The community resilience training was supported by an award from the Robert Wood Johnson Foundation. A Latina immigrant psychologist with expertise in resilience developed the training in collaboration with a community-based organization and delivered it in a small-group format to cohorts of Latino community members between 2014 and 2016 in Philadelphia, PA. The training was 40 h in total and was delivered in Spanish; participants met once a week over the course of about 4 months. Each session lasted ∼3 h. The training covered a range of topics, including resilience theory, the basic characteristics of resilient individuals, empowerment principles, and resilience for social change. The facilitator utilized a lecture format, interspersed with role-playing, videos, activities, and discussions. The training was delivered to 320 community members in total, although this number includes an unknown number of repeat participants. At the time of the training, there were no evaluation protocols in place. About a year after the conclusion of the training, researchers at Drexel University, the facilitator, and community partner who supported the training entered into a research collaboration to complete a retrospective evaluation of the training. The team deemed this evaluation important to inform the design of a future health promotion intervention for the Latino community in Philadelphia.

### Sample

For this study, we set out to recruit a convenience sample of 10 participants who took the resilience training. Given that some time had elapsed since the training, we were limited to individuals who were able to be contacted by phone and had remained in Philadelphia. Former trainees were identified by the facilitator, then contacted by our research team by phone to determine eligibility. Individuals were eligible to participate in this study if they: (1) had completed the resilience training; (2) were 18 years old or older; and (3) spoke either Spanish or English. We also interviewed the training facilitator in the study, as we were interested in capturing her perspectives. We were able to contact 12 individuals still in Philadelphia who participated in the training. Two individuals declined to participate. Ten individuals completed interviews; one participant took the training for professional reasons and was excluded from our sample since we were interested in community member perspectives for this study. We believe that saturation was reached for this study. The final sample (*N*=10) for this study consisted of nine trainees (two males and seven females) and the training facilitator herself. Participants' mean age was 39 years (standard deviation* =* 10.7); the majority were female (77%), identified as multiracial (55.6%) and Latino (90%). All participants were foreign-born, mostly from Mexico (70%). Most of them had been living in the United States for 11–20 years (60%), though the majority reported not speaking English well (70%) and not completing schooling beyond high school (80%).

### Data collection

In-depth, key informant interviews were conducted at private locations in Philadelphia. Trained, bilingual Latino interviewers conducted the interviews face-to-face by utilizing a semi-structured interview guide (Appendix 1). Interviews lasted between 45 and 90 min and were recorded by using a digital recorder. Participants received a $15 gift card for completing the interview and a short demographic survey. Interview guides were designed to inquire about participants' experiences during the resilience training, their opinions of and reactions to the training, the impact of the training on themselves and their community, use of knowledge and skills from the training, and suggestions for improvements to the training. Table 1 provides sample questions from the guide. All study procedures were approved by the Drexel University Institutional Review Board.

**Table 1.** Domains Explored in Resilience Training Participant Interviews and Selected Interview Questions—Philadelphia, PA—2017 (*N*=10)

Domains	Sample interview guide questions
Motivation to take the training	How did you learn about the training?
	What made you decide to take this training?
Novelty of resilience and community resilience	Tell me about your prior experience and knowledge about community resilience.
Relevancy of training	In general, how relevant was the training to your needs? Give us some examples of ways in which the training related to your needs?
Assessment of gains from the training	What new knowledge did you gain from the training?
	What new skills did you learn or improve on from taking the training?
Application of training content/material	After completing the training, how have you used the knowledge and skills that you learned? Give me an example.
	Have you been able to use the training you got to make changes in your community? If so, give me an example.
Personal impact of the training	Since taking the training, has something changed in the way you feel or the things you do when you are facing a problem/challenging situation? Tell me about changes in how you feel or how you behave.

### Analysis and interpretation

Digital recordings of the interviews were transcribed and translated from Spanish to English by bilingual research assistants. Data were uploaded to Dedoose analytical software, version 8.0.35. We utilized directed content analysis for this study.^21^ We first developed a codebook of *a priori* codes based on the interview guide and our research questions. Analysts then created structured summaries for each interview to become familiar with the data, which were reviewed and checked against the *a priori* codes. Two transcripts were coded by the analytical team together to promote inter-rater reliability of coding. The codebook was revised with inductive codes as needed. Transcripts were then coded by analysts individually, and coding discrepancies were resolved by group consensus. We compiled excerpts from the interviews, codes, themes, and sub-themes using analytic matrices. Working collaboratively, we integrated themes and sub-themes and developed major assertions or conclusions categorized by domains. Major themes and sub-themes that emerged during analysis are detailed in Table 2.

**Table 2.** Major Themes and Sub-Themes Identified in Participant Interviews During Analysis—Philadelphia, PA—2018

Major theme	Sub-theme
(1) Personal adversity	Past traumas affect perceptions of resilience Trauma in the past and present shape participant experiences of the training
(2) Resilience	Conceptualizing/defining resilience is deeply personal Participants recognize their own inherent strength as resilience
(3) Immigration-related stress	Participant tendency to frame resilience within the context of immigration in the United States Immigration-related stressors are the most salient for training participants
(4) Transformation of participants	Participants realize they were resilient all along Anyone can be resilient, no matter their background or what they have been through The importance of self
(5) Utility of resilience training	Resilience can be learned and should be taught to everyone Resilience can help people learn to self-regulate and cope with adversity Resilience can help others realize their own strengths and protect the community

## Results

The major assertions that summarize our findings fell under four domains: (1) Personal Pasts and Motivations; (2) Discovery of Resilience; (3) Personal Transformation; and (4) Promoting Resilience. All of these domains were mentioned by all participants in the sample, including the facilitator. See Table 3 for illustrative quotes for each domain, along with themes. These themes reflect the major assertions from our findings and are drawn from the themes and sub-themes that emerged during analyses (Table 2).

**Table 3.** Domains and Main Themes Regarding Resilience Training Experiences and Impacts: Illustrative Quotes

Domain and themes	Sample quotes
Personal pasts and motivations	
Immigrant-related adversity and other trauma	“… In this society where they exclude us, where we are in the shadows, where we cannot see our family, where we do not have the same opportunities as a citizen … I wanted to know how I could be more resilient.”—Male, 39
	“I had been through a lot of trauma. I am a survivor of incestuous rape and of domestic violence. Because of all of this I started therapy and I started doing [the training] and I was feeling very well.”—Female, 38
Discovery of resilience	
Universal nature of resilience	“It's something that all of us human beings have … it's a tool that we human beings have that we don't realize, but we have it to get ahead of adversity.”—Female, 42
	“Resilience is everywhere, in all of us, in all of our lives … from adversity to achieve a beneficial change, right?” —Male, 61
Recognizing their own resilience	“I realized that all of us [immigrants] have been resilient one way or another … it's hard at the beginning, so when you have that aspect of fighting, you look for a way. That is what I learned about resilience.”—Female, 20
	“I crossed the border, I adapted to this place and now I am learning [resilience] and I am using all my knowledge-I am using it to help others, for me it was wow!”—Female, 35
Personal transformation	
Positive sense of self	“[The training] made me understand that I had many things in me that I minimized. It made me understand that in reality I wasn't alone in spite of not having a partner, I wasn't alone. I have myself.”—Female, 38
New perspectives on trauma	“I got pregnant, the father of my daughter left me and I became depressed and when I came to take the course it made me see this another way. It made me stronger confronting things as they came and always being on my feet.”—Female, 38
Self-regulation	“Now when any situation happens to me, I try to say, ‘what am I'm going to do to move ahead?’ So that this [situation] doesn't drag me down and doesn't drown me. I know I have to pull the good out of it.”—Female, 20
Promoting resilience	
Helping and empowering others	“I try to remind them of their strengths, the potential that is in the other person. Most of us here are depressed, beaten down, victims of violence and we feel inferior to the whole world. So then, to empower the people … it's to initiate them to another world.”—Female, 45
	“Now, when I am talking to a woman and trying to help her … I can urge women to discover their own skills, talents and potential right? This makes them realize they could be women who apart from having been a victim, they also discover this other part that can empower themselves.”—Female, 33

### Personal pasts and motivations

Participants were highly motivated to take part in the resilience training because they viewed it as responsive to their individual needs and interests. Participants were drawn to the training because of the hardships and trauma they had endured—much of it immigration related. These experiences affected their mental and emotional health. The training facilitator highlighted unmet mental health needs among Latino immigrants in Philadelphia. She felt that participants' motivation to be trained in resilience stemmed from social inequity and injustice. Despite prolonged exposure to hardship during their immigrant journeys, immigrants are left to tend to their psychological health in the United States, even as they struggle to assimilate. Participants related experiences of immigration-related adversity, such as racism and social exclusion, as well as other traumatic experiences such as rape and domestic violence. Participants' motivation to take the training also included the need to overcome a sense of isolation. Belonging to a community and being able to access social support and other interpersonal resources was of great importance for those who described feeling “alone” or “a nobody.” Participants described various reasons for training in resilience, but most converged on their past traumas and ongoing struggles as immigrants.

### Discovery of resilience

Participants recognized their own resilience and developed their own conceptualizations of resilience. Resilience was a novel concept for trainees, most of whom had never heard of the term. Despite being unfamiliar with resilience before the training, participants were quick to relate the theories and concepts of resilience to their own lives. During her interview, the training facilitator defined resilience as “the ability of all human beings to face adversities and get ahead of their lives.” Her definition resonated with trainees, who described resilience by using similar words and terms. They spoke about the universal nature of resilience in all humanity, in addition to resilience as a “tool” to confront adversity. Participants also reported discovering their own personal resilience as part of the training, which some equated with the will to fight and survive. They associated being resilient with being an immigrant enduring societal oppression, while at the same time conceptualizing resilience as transcending national borders, citizenship, and other sociopolitical structures.

### Personal transformation

The training was personally transformative for participants in that it helped them redefine their sense of self and gain new, healthier perspectives of their lives. Several trainees described realizing during the training that they had been resilient all along, having endured traumas in their pasts and daily adversity in this country. Recognizing their own personal strength and power was transformative. This transformation included a more positive sense of self. Newfound awareness of their inner strengths catalyzed this re-definition of self by instilling in them a renewed sense of their own ability to carry on. Many spoke about changes in how they view traumatic experiences. Before the training, some participants felt bad about themselves because of their traumas; re-defining themselves as resilient survivors helped attenuate these feelings of guilt and self-deprecation. The training also helped participants to more effectively cope with day-to-day adversity. It enhanced their capacity to emotionally self-regulate and likely contributed to improved mental/emotional health. Self-regulation, coupled with a reconstructed sense of self, resulted in a more positive outlook on life. Participants described how they learned to live in the moment, enjoy the beauty of life, and take care of themselves. One participant told us that she learned to “be more thankful for life, to value [her]self, to spoil [her]self, to take time for [her]self.” She explained that by shoring up herself, she can better care for others, such as her own children. Indeed, several of our female participants discussed improvements in parenting and more meaningful relationships with family and friends, which they associated with the knowledge and skills obtained from the training.

### Promoting resilience

Participants used the skills and knowledge from the training to help others become more resilient. The impact of the training extended beyond participants as individuals. They recounted using the knowledge and skills they acquired from the training to help others in their networks. They were able to help peers tap into and become more aware of their resilience through conversations that focused on “help[ing] others help themselves.” They worked to empower others by using resilience teachings to support their peers through hardship by offering advice and support.

All participants reported talking to at least one person about resilience after the training. Having become aware of their own resilience, they were eager to awaken this knowledge in others in their networks via one-on-one interactions. Other participants, though they felt the urge to promote resilience, were less confident in their abilities to do so; reasons cited included not being very “active” in their communities or just feeling that their peers did not understand resilience or viewed it as unimportant.

## Discussion

This study examined participants' reactions to a resilience-focused program for Latino immigrants implemented in Philadelphia. The results explore the acceptability and potential of resilience-based interventions to promote Latino health and well-being. The participants found that the training resonated deeply with them because of their personal histories of trauma and adversity. Resilience is more than the absence of psychopathology.^22^ Core characteristics of resilient individuals include an internal locus of control, commitment to self, optimism,^23^ and supporting others.^24,25^ These factors emerged in participants' narratives. Trainees described feeling more self-reliant, being more committed to self-care, viewing their lives more positively, and even working to strengthen resilience in others. We believe that the training fostered or enhanced a sense of resilience among participants, who felt better equipped to cope with past, current, and future challenges. Resilience is a powerful and positive force for coping and it has been shown to help alleviate feelings of helplessness amid misfortune.^23^ This protective effect was echoed by our participants, who described being more aware of their own strength and ability to regulate their emotions as a result of the training. To our knowledge, no other evaluation of a resilience training for Latino immigrant adults has been published. However, evaluations of resilience trainings for other adult and youth groups indicate that resilience building mitigates the development of mental health issues after trauma, increases empathy and self-esteem, and assists in the development of self-regulation skills.^25–27^ Our findings support this developing literature.

Many participants recounted immigration-related adversity, such as feeling excluded from society, being unable to seek help or services, and being targeted by law enforcement. Their understandings of resilience were shaped by these unique challenges. We posit that a resilience approach could be utilized to talk about and frame adversity related to being a Latino immigrant in the United States, particularly in today's increasingly anti-immigrant climate. Studies have documented the challenges that Latino immigrant adults face as they acclimate to life in the United States.^28^ Individually, immigrants are likely to be handicapped by factors such as low education, English language barriers, and discrimination.^28,29^ However, research suggests the need to go beyond individual-level interventions in an effort to build community resources, such as strengthening within-group ties and connecting immigrants with the broader society.^29^ In addition, research suggests that there are multiple pathways to resilience.^21^ Although some studies have classified Latino populations as resilient, it is important to note that very few studies have explored factors contributing to increased resilience as well as risk factors that may erode it.^14,23^ Future research is needed to delineate the specific pathways through which resilience-based interventions can help to fortify Latino communities to overcome crises, especially among adults, where research is particularly lacking.^14,21^ Studies of resilient groups can provide valuable insight into the true nature of resilience and its relationship to health and well-being.^27^

Participants described promoting resilience within their networks, even though the training curriculum did not include a formal community outreach component. Many reported talking to others about resilience explicitly, as well as simply working to support them through hardship. These actions increased feelings of connectedness, which is critical in resilience models.^15,30^ The resilience framework could be linked to existing evidence-based approaches that rely on community members.^31^ Peers can work to create more resilient communities. Such communities, in turn, provide opportunities and conditions that enable people to better cope with problems as they arise.^32,33^ Our findings suggest that programs that strengthen individual resilience may contribute to create resilient communities, provided that participants are confident and knowledgeable about how to promote resilience. Resilient Latino community members can act as agents of change to help address factors that directly affect Latino health, by helping peers to identify their own resilience, develop coping skills, and maintain cultural and community ties. This evaluation suggests that resilience can enhance interventions for Latino immigrant adults, especially community-based models prioritizing community resilience as a protective factor for health.^34,35^

## Limitations

There are important limitations of our study that should be noted. Participants were asked to recall details of a training they had completed a few years earlier. It is possible that our findings are subject to recall bias, since participants might not have recalled their experiences accurately or completely. The convenient and small sample (*N*=10) limits the generalizability of the findings, although generalizability is not necessarily the primary goal of qualitative research.^21^ However, the pool of participants was limited to those who researchers were able to contact; thus, they may represent a special subset of individuals whose opinions may not be shared with the larger group of resilience training participants. Finally, participants may have felt compelled to report positive opinions of the training. Participants were reassured that the information they provided would not be linked to their identity and this may have reduced such bias.

## Health Equity Implications

A resilience approach is valuable for public health since it supports the development of strong communities.^6^ It is an effective and acceptable tool with which to strengthen individuals and their communities against adversity, including social and health inequities, as well as to promote mental health. A resilience model can help to increase social capital and reduce isolation, which, in turn, may have a protective effect on mental health outcomes and should be considered by mental health practitioners and researchers as a valid approach to promote psychological well-being. Further, resilience cannot and should not be separated from individual and community-level trauma and should receive more attention from public health practitioners, activists, and policy makers. Finally, our findings suggest that resilience in Latino immigrants can be protective of psychosocial health by empowering individuals to better cope with immigrant-related challenges.^16,36^ Our study findings highlight the need for further research on interventions that build resilience, as a stand-alone approach and as an additional element in other evidence-based approaches. It is probable that resilience can be harnessed to enhance coordinated efforts to reduce Latino immigrant health disparities by pushing for social change with a grassroots spirit.

## Informed Consent

Informed consent was obtained from all individual participants included in the study.

## Ethical Approval

All procedures performed in studies involving human participants were in accordance with the ethical standards of the institutional and/or national research committee and with the 1964 Helsinki declaration and its later amendments or comparable ethical standards.

## Appendix 1

### Community Resilience Training Participant Semi-Structured Interview Guide

Thank you for your time today. We're collaborating with TPAC to assess the Community Resilience Training that you were part of. Your contribution to this assessment will help adapt the training, content, and delivery to better serve the needs of the community.

1. When did you complete this training?
2. Reason/motivation for taking training:
2. How did you learn about the training?
3. What made you decide to take this training?
4. How motivated were you to learn about community resilience and the different topics presented when you first attended this training?
6. Prior experience/knowledge and training:
5. Tell me about your prior experience and knowledge about Community Resilience.
6. Have you received any previous training related to this topic?
6. 1. When, where?
6. 2. How did this training at TPAC compare with previous Community Resilience trainings?
11. Format and methods: Let's talk about how the training was done
7. First of all, tell me what the training consisted of and how it was delivered, as you remember it now.
7. 1. Interviewer: Probe for group activities, group discussions, lectures, exercises, and assignments. Probe for one session, multiple sessions, etc.
8. [Interviewer: Depending on the format that the training used, phrase the next question]. From the lectures and activities (ex. group activities, group discussions, etc) that were used by the training facilitator, what was/were the most helpful to you? Why?
9. Which training method was the least helpful to you? Why?
10. Was the length of the training appropriate? Why/why not? (total length of course, length of each session?)
17. Assessment of content: Let's talk about the content/topics of the training.
11. Can you tell me whether the content was easy to understand or difficult to understand? What made it easy/difficult to understand?
12. Which topic/topics were the most interesting to you? Why?
13. Which topic/topics were the least interesting to you? Why?
14. Did the facilitator use materials (such as handouts, videos, PowerPoint slides) during the training? (yes/no)—if so, which materials helped you the most? Which were the least helpful?
22. General relevance:
15. In general, how relevant was the training to your needs? Give us some examples of ways in which the training related to your needs.
16. What did you expect to gain from this training? (e.g. any knowledge or skills you wanted to gain)
17. In general, how did the training meet your learning expectations? Why?
26. General satisfaction:
18. In general, how satisfied did you feel about the training? If you had to rate your level of satisfaction as low, medium, high, or excellent, how would you rate it? Why?
28. Assessment of learning and maintenance:
29. Ok, finally, I'd like to talk about the things you learned in the CR training and how you may have used what you learned since you took the training.
19. What new knowledge did you gain from the training?
20. What new skills did you learn/or improve on from taking the training?
21. After completing the training, how have you used the knowledge and skills that you learned? (if knowledge and skills were reported under assessment of learning) Give me an example.
22. Since taking the training, has something changed in the way you feel or the things you do when [you are facing / you are helping a client who is facing] a problem/challenging situation? Tell me about changes in how you feel or how you behave.
23. Have you been able to use the training you got to make changes in your organization, community? If so, how? or give me an example.
24. Do you continue to use anything you learned in the training?
24. 1. [If yes] Tell me more about the things you still use and why do you still use them.
24. 2. Are there things you learned that you don't use much these days?
24. 3. [If participant does not use what was learned in training] Why not? What would help you use the things you learned?
39. Suggestion for improvements:
40. Now, I'd like to know about your thoughts and ideas for making the training better
25. Do you have any suggestions for making the training better? What changes would you suggest? (Interviewer: Probe for changes in content, delivery, facilitator, venue).
